# Supplementary material for: Exploring Dengue Dynamics: A Multi-Scale Analysis of Spatio-Temporal Trends in Ibagué, Colombia
Source: Viruses. 2024 Jun 3;16(6):906. doi: 10.3390/v16060906 (PMC11209037; doi:10.3390/v16060906)
Supplement: Supplementary file 1 [file viruses-16-00906-s001.zip › viruses-2931885-supplementary.pdf]

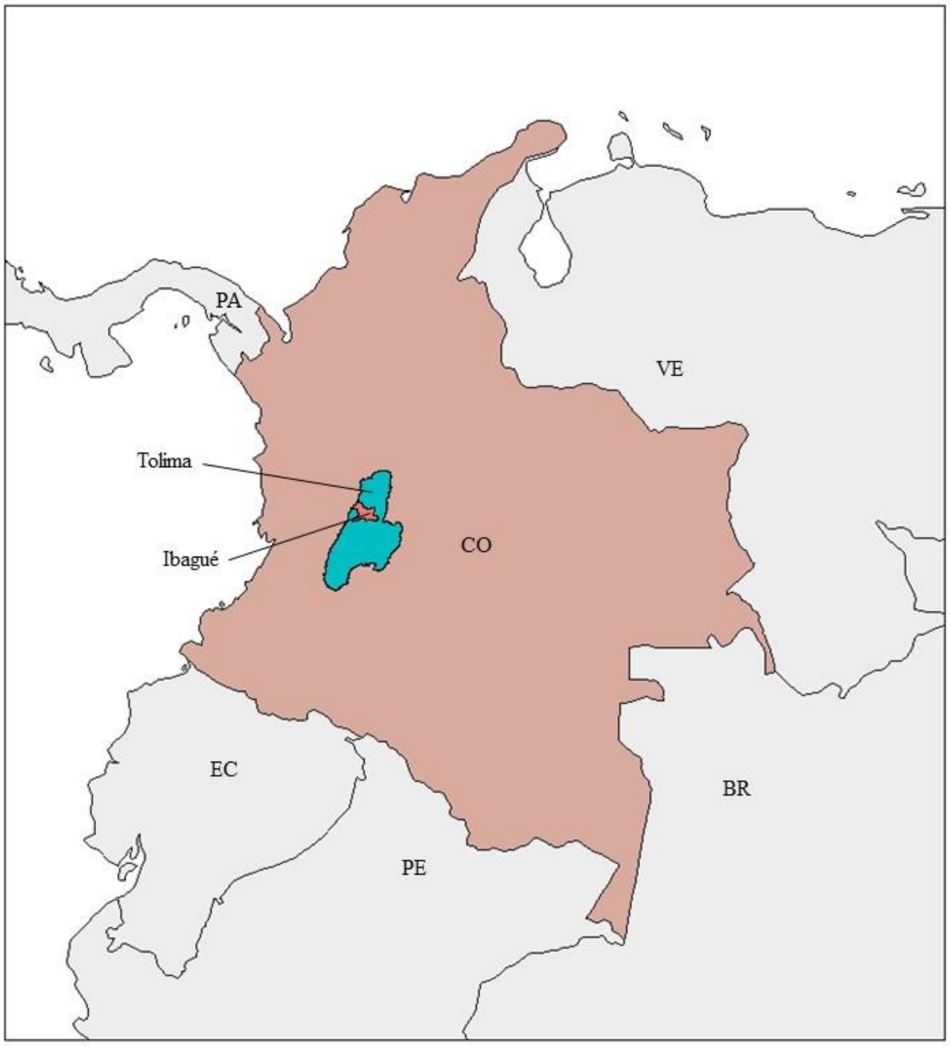

Figure S1. Ibague's location in the department of Tolima, Colombia (CO) and neighboring countries including Venezuela (VE), Brazil (BR), Peru (PE), Ecuador (EC) and Panama (PA).

Table S1. Details about aggregation levels

	Range			
**Unit information**	**Comunas**	**Sectores**	**Secciones**	**Manzanas**
Area [km^2]	1.24 – 10.78	0.07 – 8.25	0.01– 1.86	5.83E-05 – 0.84
Number of blocks per unit	169 - 862	2 - 352	1 - 87	1
Total number of units	13	63	272	5,574

Table S2. List of independent socioeconomic, demographic, and environmental variables for Ibagué.

					Range			
**Variable**	**Category**	**Description**	**Resolution**	**Source**	**Comunas**	**Sectores**	**Secciones**	**Manzanas**
Population	Demographic	Count of people	Block	DANE	14,903 – 71,226	94 – 25,062	0 – 6345	0 – 2,946
Density	Demographic	Population density [ppl/km^2]	Block	DANE	5,983 – 23,584	569 – 36,759	0 – 66,160	0 – 378,700
Houses	Demographic	Count of houses	Block	DANE	6,241 – 26,895	34 – 12,178	0 – 3,116	0 – 1,075
Homes	Demographic	Count of populated houses	Block	DANE	5,221 – 24,464	31 – 8,640	0 – 2,205	0 – 1,071
Electricity	Socioeconomic	Percentage of houses with electricity	Block	DANE	0.77 – 0.95	0.64 – 0.98	0 – 1	0 – 1
Aqueduct	Socioeconomic	Percentage of houses connected to the aqueduct	Block	DANE	0.76 – 0.92	0.64 – 0.97	0 – 1	0 – 1
Sewage	Socioeconomic	Percentage of houses connected to the sewage	Block	DANE	0.76 – 0.94	0.03 – 0.97	0 – 1	0 – 1
Gas	Socioeconomic	Percentage of houses connected to the gas network	Block	DANE	0.69 – 0.90	0.49 – 0.94	0 – 0.96	0 – 1
Garbage	Socioeconomic	Percentage of houses with garbage pickup service	Block	DANE	0.76 – 0.94	0.63 – 0.99	0 – 1	0 – 1
Internet	Socioeconomic	Percentage of houses with internet connection	Block	DANE	0.28 – 0.67	0.09 – 0.75	0 – 0.84	0 – 1
Strata	Socioeconomic	Average strata	Block	DANE	1 – 3	1 – 3	0 – 3	0 – 5
Higher Ed.	Demographic	Percentage of people with a higher education degree (after high school)	Block	DANE	0.12 – 0.40	0.06 – 0.58	0 – 0.61	0 – 0.86
No Ed.	Demographic	Percentage of people without any education	Block	DANE	0.02 – 0.04	0 – 0.06	0 – 0.08	0 – 0.48
Kids	Demographic	Percentage of people under 19 years old	Block	DANE	0.23 – 0.34	0.15 – 0.37	0 – 0.41	0 – 0.58
Adults	Demographic	Percentage of people over 19 years old	Block	DANE	0.66 – 0.77	0.63 – 0.85	0 – 0.88	0 – 1
Men	Demographic	Percentage of people identifying as man	Block	DANE	0.46 – 0.48	0.44 – 0.52	0 – 0.62	0 – 0.93
Women	Demographic	Percentage of people identifying as woman	Block	DANE	0.51 – 0.54	0.48 – 0.56	0 – 0.62	0 – 0.81
Total Prec.	Environmental	Total precipitation [mm/month]	1 km	CHIRPS	0.81 – 118	0.05 – 89.78	0.01 – 20.36	0 – 9.25
Mean Temp.	Environmental	Mean temperature during each month [°C]	0.05°	MODIS	22.96 – 38.77	21.98 – 39.62	21.03 – 39.75	20.51 – 39.75
Wet Days	Environmental	Count of raining days each month	City	-	2 – 20	2 – 20	2 – 20	2- 20
Days ov. 32	Environmental	Count of days when the maximum temperature exceeded 32°C each month	City	-	0 – 29	0 – 29	0 – 29	0 – 29
NDVI	Environmental	Normalized Vegetation Index per month	0.25 km	MODIS	0.23 – 0.71	0.16 – 0.80	0.19 – 0.87	0.14 – 0.90

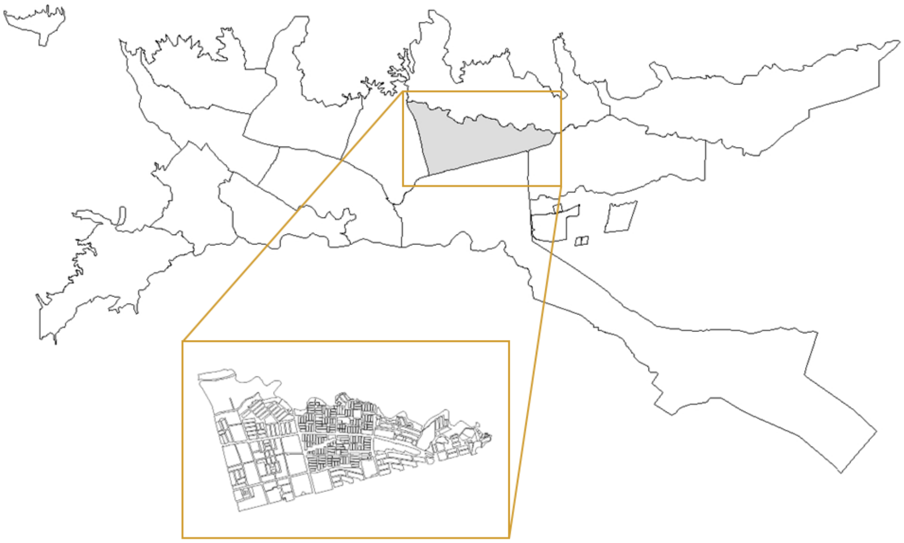

Figure S2. Ibague's spatial division, from which aggregation levels are obtained. Comunas are the biggest group, each composed of multiple manzanas as shown in the figure. Smaller groups of manzanas build secciones and sectores.

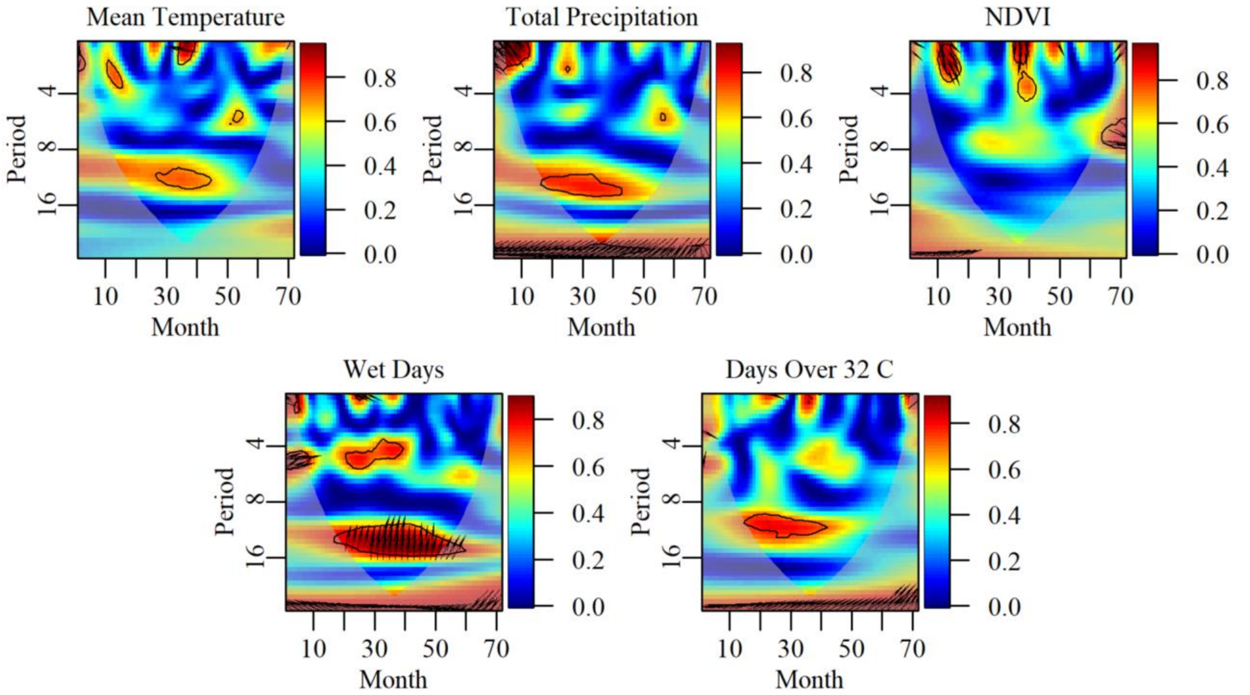

Figure S3. Wavelet coherence analysis for environmental variables. Each plot shows the coherence between the environmental variables and DENV cases. High-significance (0.05) regions are plotted as bounded areas. A cone of influence is included to discard any significant regions outside it. Arrows indicate the relationship of the two series included in each plot.
